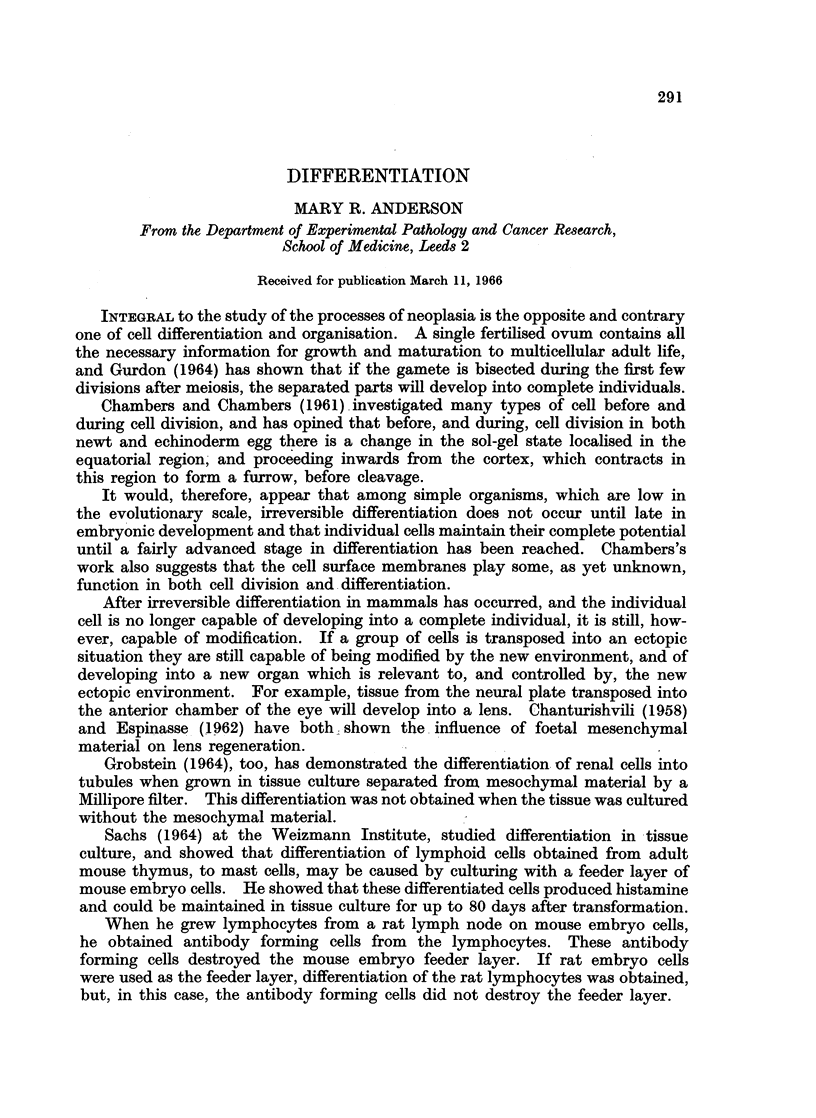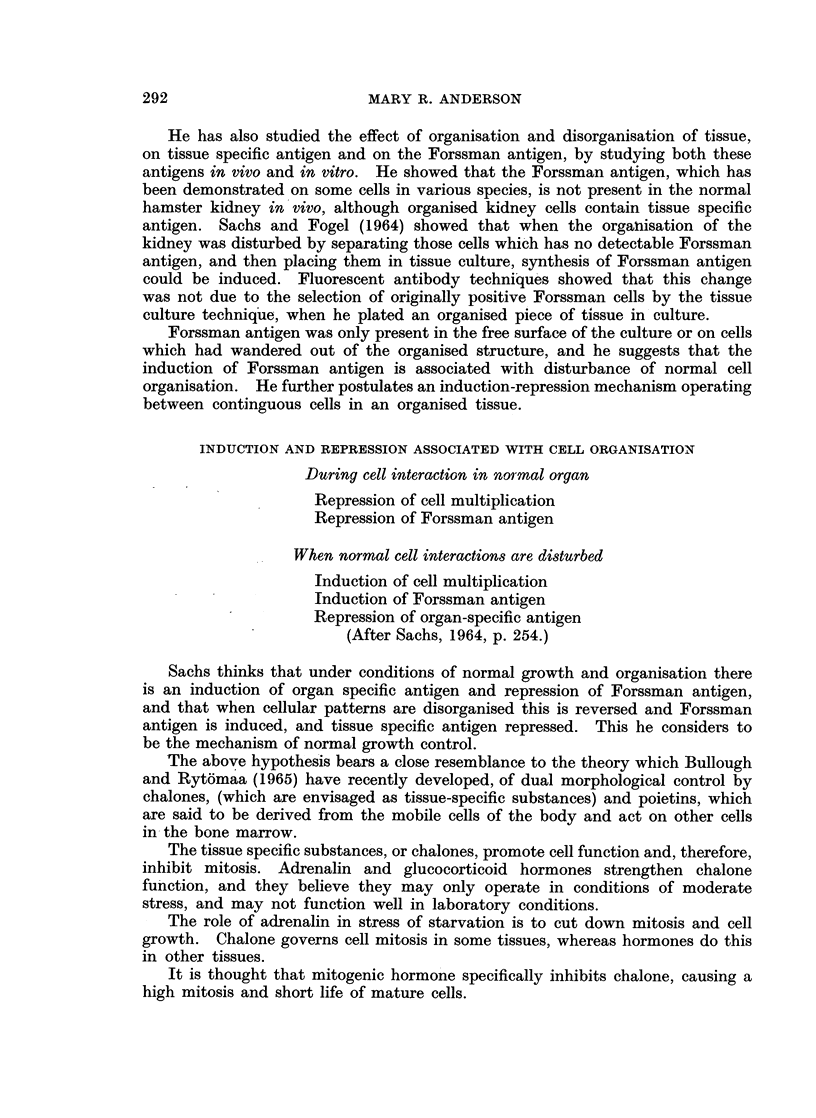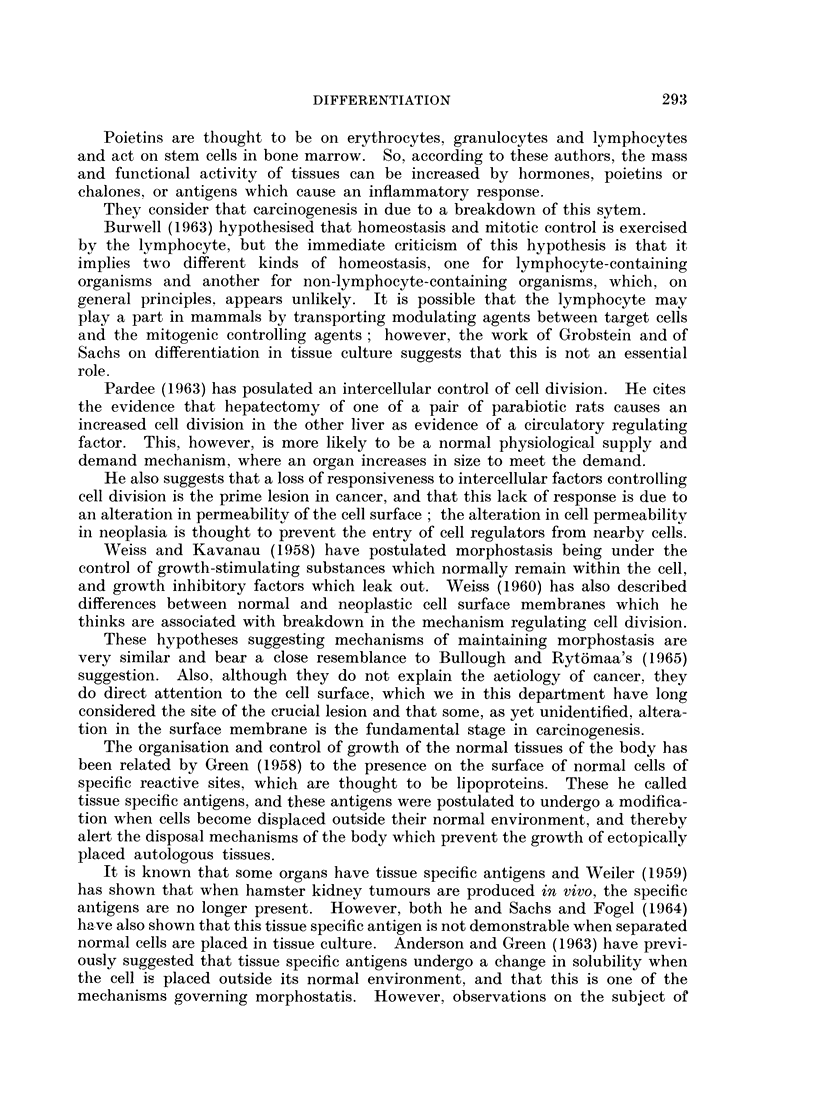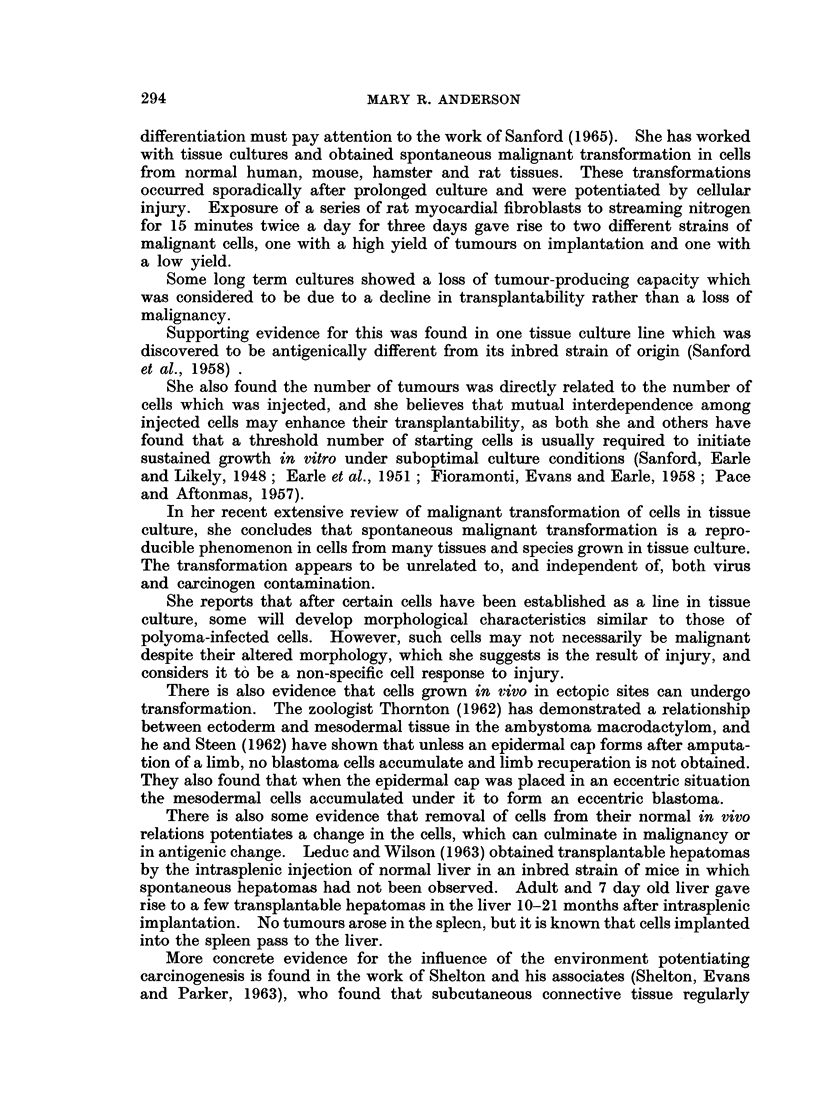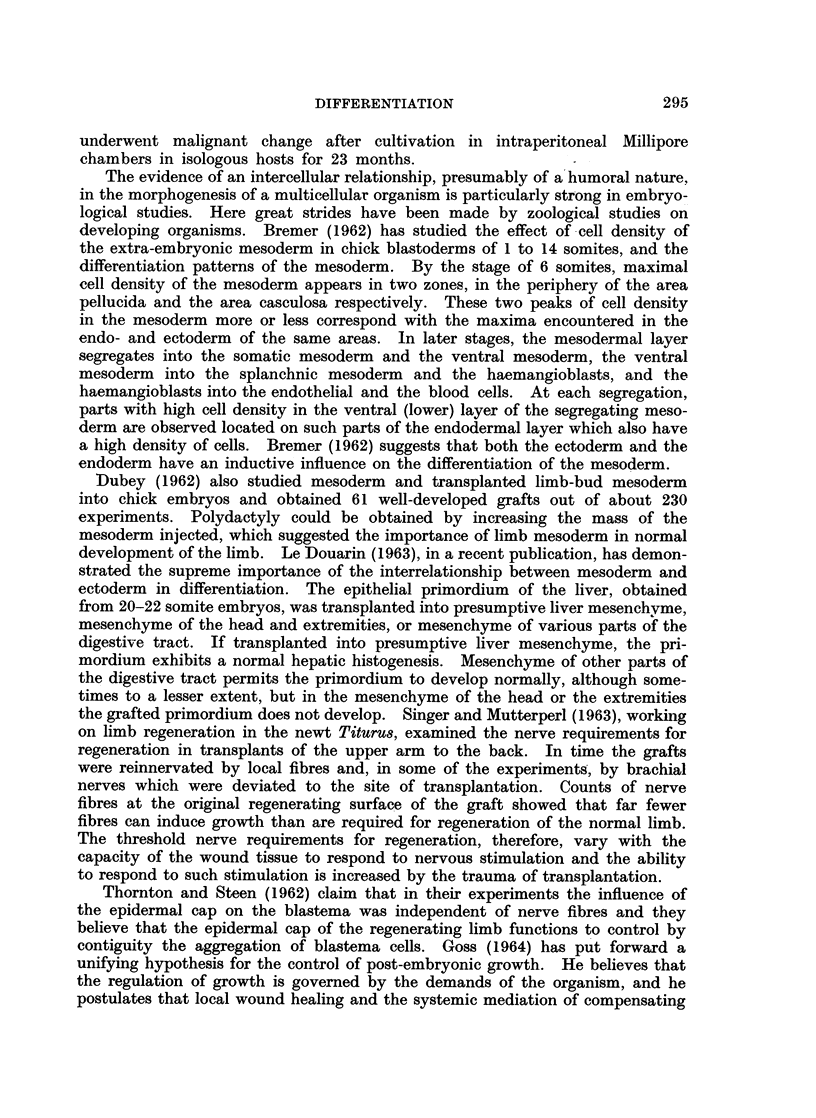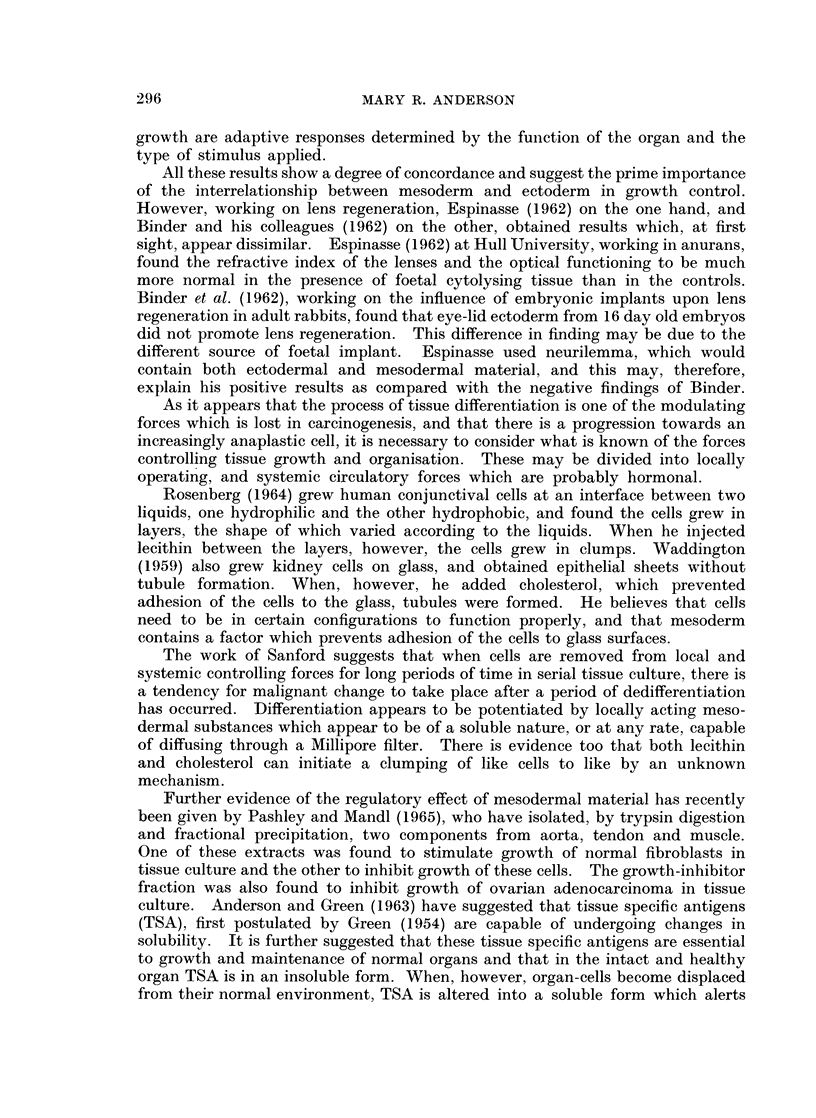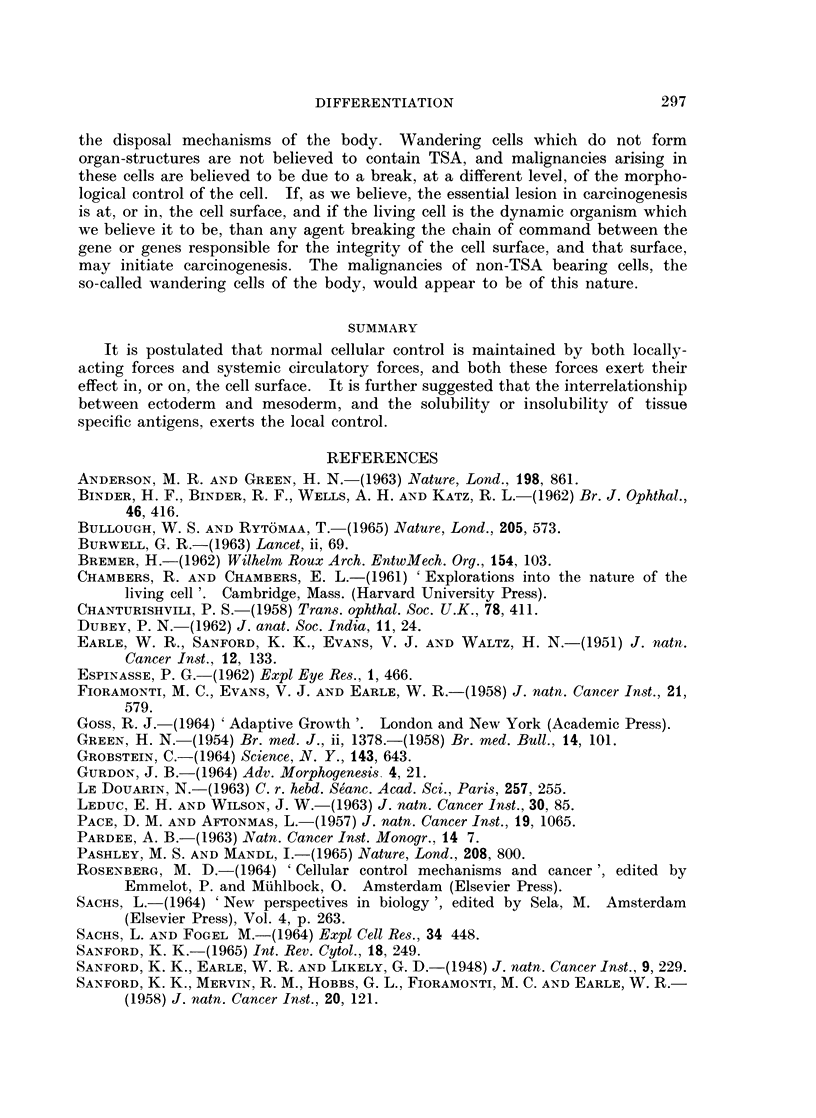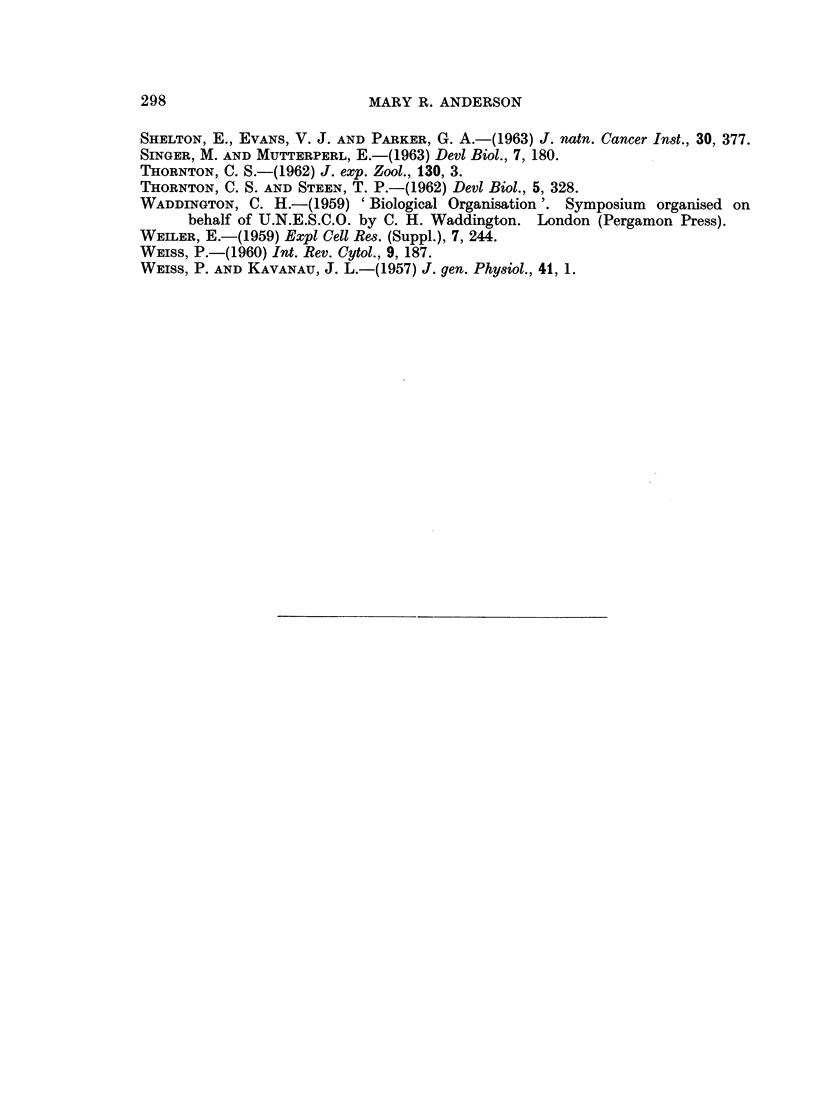# Differentiation.

**DOI:** 10.1038/bjc.1966.37

**Published:** 1966-06

**Authors:** M. R. Anderson


### DIFFERENTIATION

MARY R. ANDERSON

*From the Department of Experimental Pathology and Cancer Research, School of Medicine, Leeds 2*

Received for publication March 11, 1966

INTEGRAL to the study of the processes of neoplasia is the opposite and contrary one of cell differentiation and organisation. A single fertilised ovum contains all the necessary information for growth and maturation to multicellular adult life, and Gurdon (1964) has shown that if the gamete is bisected during the first few divisions after meiosis, the separated parts will develop into complete individuals.

Chambers and Chambers (1961) investigated many types of cell before and during cell division, and has opined that before, and during, cell division in both newt and echinoderm egg there is a change in the sol-gel state localised in the equatorial region, and proceeding inwards from the cortex, which contracts in this region to form a furrow, before cleavage.

It would, therefore, appear that among simple organisms, which are low in the evolutionary scale, irreversible differentiation does not occur until late in embryonic development and that individual cells maintain their complete potential until a fairly advanced stage in differentiation has been reached. Chambers's work also suggests that the cell surface membranes play some, as yet unknown, function in both cell division and differentiation.

After irreversible differentiation in mammals has occurred, and the individual cell is no longer capable of developing into a complete individual, it is still, however, capable of modification. If a group of cells is transposed into an ectopic situation they are still capable of being modified by the new environment, and of developing into a new organ which is relevant to, and controlled by, the new ectopic environment. For example, tissue from the neural plate transposed into the anterior chamber of the eye will develop into a lens. Chanturishvili (1958) and Espinasse (1962) have both shown the influence of foetal mesenchymal material on lens regeneration.

Grobstein (1964), too, has demonstrated the differentiation of renal cells into tubules when grown in tissue culture separated from mesochymal material by a Millipore filter. This differentiation was not obtained when the tissue was cultured without the mesochymal material.

Sachs (1964) at the Weizmann Institute, studied differentiation in tissue culture, and showed that differentiation of lymphoid cells obtained from adult mouse thymus, to mast cells, may be caused by culturing with a feeder layer of mouse embryo cells. He showed that these differentiated cells produced histamine and could be maintained in tissue culture for up to 80 days after transformation.

When he grew lymphocytes from a rat lymph node on mouse embryo cells, he obtained antibody forming cells from the lymphocytes. These antibody forming cells destroyed the mouse embryo feeder layer. If rat embryo cells were used as the feeder layer, differentiation of the rat lymphocytes was obtained, but, in this case, the antibody forming cells did not destroy the feeder layer.

He has also studied the effect of organisation and disorganisation of tissue, on tissue specific antigen and on the Forssman antigen, by studying both these antigens *in vivo* and *in vitro*. He showed that the Forssman antigen, which has been demonstrated on some cells in various species, is not present in the normal hamster kidney *in vivo*, although organised kidney cells contain tissue specific antigen. Sachs and Fogel (1964) showed that when the organisation of the kidney was disturbed by separating those cells which has no detectable Forssman antigen, and then placing them in tissue culture, synthesis of Forssman antigen could be induced. Fluorescent antibody techniques showed that this change was not due to the selection of originally positive Forssman cells by the tissue culture technique, when he plated an organised piece of tissue in culture.

Forssman antigen was only present in the free surface of the culture or on cells which had wandered out of the organised structure, and he suggests that the induction of Forssman antigen is associated with disturbance of normal cell organisation. He further postulates an induction-repression mechanism operating between continguous cells in an organised tissue.

INDUCTION AND REPRESSION ASSOCIATED WITH CELL ORGANISATION

*During cell interaction in normal organ*

Repression of cell multiplication
Repression of Forssman antigen

*When normal cell interactions are disturbed*

Induction of cell multiplication
Induction of Forssman antigen
Repression of organ-specific antigen
(After Sachs, 1964, p. 254.)

Sachs thinks that under conditions of normal growth and organisation there is an induction of organ specific antigen and repression of Forssman antigen, and that when cellular patterns are disorganised this is reversed and Forssman antigen is induced, and tissue specific antigen repressed. This he considers to be the mechanism of normal growth control.

The above hypothesis bears a close resemblance to the theory which Bullough and Rytömaa (1965) have recently developed, of dual morphological control by chalones, (which are envisaged as tissue-specific substances) and poietins, which are said to be derived from the mobile cells of the body and act on other cells in the bone marrow.

The tissue specific substances, or chalones, promote cell function and, therefore, inhibit mitosis. Adrenalin and glucocorticoid hormones strengthen chalone function, and they believe they may only operate in conditions of moderate stress, and may not function well in laboratory conditions.

The role of adrenalin in stress of starvation is to cut down mitosis and cell growth. Chalone governs cell mitosis in some tissues, whereas hormones do this in other tissues.

It is thought that mitogenic hormone specifically inhibits chalone, causing a high mitosis and short life of mature cells.

Poietins are thought to be on erythrocytes, granulocytes and lymphocytes and act on stem cells in bone marrow. So, according to these authors, the mass and functional activity of tissues can be increased by hormones, poietins or chalones, or antigens which cause an inflammatory response.

They consider that carcinogenesis in due to a breakdown of this sytem.

Burwell (1963) hypothesised that homeostasis and mitotic control is exercised by the lymphocyte, but the immediate criticism of this hypothesis is that it implies two different kinds of homeostasis, one for lymphocyte-containing organisms and another for non-lymphocyte-containing organisms, which, on general principles, appears unlikely. It is possible that the lymphocyte may play a part in mammals by transporting modulating agents between target cells and the mitogenic controlling agents ; however, the work of Grobstein and of Sachs on differentiation in tissue culture suggests that this is not an essential role.

Pardee (1963) has posulated an intercellular control of cell division. He cites the evidence that hepatectomy of one of a pair of parabiotic rats causes an increased cell division in the other liver as evidence of a circulatory regulating factor. This, however, is more likely to be a normal physiological supply and demand mechanism, where an organ increases in size to meet the demand.

He also suggests that a loss of responsiveness to intercellular factors controlling cell division is the prime lesion in cancer, and that this lack of response is due to an alteration in permeability of the cell surface ; the alteration in cell permeability in neoplasia is thought to prevent the entry of cell regulators from nearby cells.

Weiss and Kavanau (1958) have postulated morphostasis being under the control of growth-stimulating substances which normally remain within the cell, and growth inhibitory factors which leak out. Weiss (1960) has also described differences between normal and neoplastic cell surface membranes which he thinks are associated with breakdown in the mechanism regulating cell division.

These hypotheses suggesting mechanisms of maintaining morphostasis are very similar and bear a close resemblance to Bullough and Rytömaa's (1965) suggestion. Also, although they do not explain the aetiology of cancer, they do direct attention to the cell surface, which we in this department have long considered the site of the crucial lesion and that some, as yet unidentified, alteration in the surface membrane is the fundamental stage in carcinogenesis.

The organisation and control of growth of the normal tissues of the body has been related by Green (1958) to the presence on the surface of normal cells of specific reactive sites, which are thought to be lipoproteins. These he called tissue specific antigens, and these antigens were postulated to undergo a modification when cells become displaced outside their normal environment, and thereby alert the disposal mechanisms of the body which prevent the growth of ectopically placed autologous tissues.

It is known that some organs have tissue specific antigens and Weiler (1959) has shown that when hamster kidney tumours are produced *in vivo*, the specific antigens are no longer present. However, both he and Sachs and Fogel (1964) have also shown that this tissue specific antigen is not demonstrable when separated normal cells are placed in tissue culture. Anderson and Green (1963) have previously suggested that tissue specific antigens undergo a change in solubility when the cell is placed outside its normal environment, and that this is one of the mechanisms governing morphostatis. However, observations on the subject of differentiation must pay attention to the work of Sanford (1965). She has worked with tissue cultures and obtained spontaneous malignant transformation in cells from normal human, mouse, hamster and rat tissues. These transformations occurred sporadically after prolonged culture and were potentiated by cellular injury. Exposure of a series of rat myocardial fibroblasts to streaming nitrogen for 15 minutes twice a day for three days gave rise to two different strains of malignant cells, one with a high yield of tumours on implantation and one with a low yield.

Some long term cultures showed a loss of tumour-producing capacity which was considered to be due to a decline in transplantability rather than a loss of malignancy.

Supporting evidence for this was found in one tissue culture line which was discovered to be antigenically different from its inbred strain of origin (Sanford *et al.*, 1958) .

She also found the number of tumours was directly related to the number of cells which was injected, and she believes that mutual interdependence among injected cells may enhance their transplantability, as both she and others have found that a threshold number of starting cells is usually required to initiate sustained growth *in vitro* under suboptimal culture conditions (Sanford, Earle and Likely, 1948 ; Earle *et al.*, 1951 ; Fioramonti, Evans and Earle, 1958 ; Pace and Aftonmas, 1957).

In her recent extensive review of malignant transformation of cells in tissue culture, she concludes that spontaneous malignant transformation is a reproducible phenomenon in cells from many tissues and species grown in tissue culture. The transformation appears to be unrelated to, and independent of, both virus and carcinogen contamination.

She reports that after certain cells have been established as a line in tissue culture, some will develop morphological characteristics similar to those of polyoma-infected cells. However, such cells may not necessarily be malignant despite their altered morphology, which she suggests is the result of injury, and considers it to be a non-specific cell response to injury.

There is also evidence that cells grown *in vivo* in ectopic sites can undergo transformation. The zoologist Thornton (1962) has demonstrated a relationship between ectoderm and mesodermal tissue in the ambystoma macrodactylom, and he and Steen (1962) have shown that unless an epidermal cap forms after amputation of a limb, no blastoma cells accumulate and limb recuperation is not obtained. They also found that when the epidermal cap was placed in an eccentric situation the mesodermal cells accumulated under it to form an eccentric blastoma.

There is also some evidence that removal of cells from their normal *in vivo* relations potentiates a change in the cells, which can culminate in malignancy or in antigenic change. Leduc and Wilson (1963) obtained transplantable hepatomas by the intrasplenic injection of normal liver in an inbred strain of mice in which spontaneous hepatomas had not been observed. Adult and 7 day old liver gave rise to a few transplantable hepatomas in the liver 10–21 months after intrasplenic implantation. No tumours arose in the spleen, but it is known that cells implanted into the spleen pass to the liver.

More concrete evidence for the influence of the environment potentiating carcinogenesis is found in the work of Shelton and his associates (Shelton, Evans and Parker, 1963), who found that subcutaneous connective tissue regularly underwent malignant change after cultivation in intraperitoneal Millipore chambers in isologous hosts for 23 months.

The evidence of an intercellular relationship, presumably of a humoral nature, in the morphogenesis of a multicellular organism is particularly strong in embryological studies. Here great strides have been made by zoological studies on developing organisms. Bremer (1962) has studied the effect of cell density of the extra-embryonic mesoderm in chick blastoderms of 1 to 14 somites, and the differentiation patterns of the mesoderm. By the stage of 6 somites, maximal cell density of the mesoderm appears in two zones, in the periphery of the area pellucida and the area casculosa respectively. These two peaks of cell density in the mesoderm more or less correspond with the maxima encountered in the endo- and ectoderm of the same areas. In later stages, the mesodermal layer segregates into the somatic mesoderm and the ventral mesoderm, the ventral mesoderm into the splanchnic mesoderm and the haemangioblasts, and the haemangioblasts into the endothelial and the blood cells. At each segregation, parts with high cell density in the ventral (lower) layer of the segregating mesoderm are observed located on such parts of the endodermal layer which also have a high density of cells. Bremer (1962) suggests that both the ectoderm and the endoderm have an inductive influence on the differentiation of the mesoderm.

Dubey (1962) also studied mesoderm and transplanted limb-bud mesoderm into chick embryos and obtained 61 well-developed grafts out of about 230 experiments. Polydactyly could be obtained by increasing the mass of the mesoderm injected, which suggested the importance of limb mesoderm in normal development of the limb. Le Douarin (1963), in a recent publication, has demonstrated the supreme importance of the interrelationship between mesoderm and ectoderm in differentiation. The epithelial primordium of the liver, obtained from 20–22 somite embryos, was transplanted into presumptive liver mesenchyme, mesenchyme of the head and extremities, or mesenchyme of various parts of the digestive tract. If transplanted into presumptive liver mesenchyme, the primordium exhibits a normal hepatic histogenesis. Mesenchyme of other parts of the digestive tract permits the primordium to develop normally, although sometimes to a lesser extent, but in the mesenchyme of the head or the extremities the grafted primordium does not develop. Singer and Mutterperl (1963), working on limb regeneration in the newt *Titurus*, examined the nerve requirements for regeneration in transplants of the upper arm to the back. In time the grafts were reinnervated by local fibres and, in some of the experiments, by brachial nerves which were deviated to the site of transplantation. Counts of nerve fibres at the original regenerating surface of the graft showed that far fewer fibres can induce growth than are required for regeneration of the normal limb. The threshold nerve requirements for regeneration, therefore, vary with the capacity of the wound tissue to respond to nervous stimulation and the ability to respond to such stimulation is increased by the trauma of transplantation.

Thornton and Steen (1962) claim that in their experiments the influence of the epidermal cap on the blastema was independent of nerve fibres and they believe that the epidermal cap of the regenerating limb functions to control by contiguity the aggregation of blastema cells. Goss (1964) has put forward a unifying hypothesis for the control of post-embryonic growth. He believes that the regulation of growth is governed by the demands of the organism, and he postulates that local wound healing and the systemic mediation of compensating growth are adaptive responses determined by the function of the organ and the type of stimulus applied.

All these results show a degree of concordance and suggest the prime importance of the interrelationship between mesoderm and ectoderm in growth control. However, working on lens regeneration, Espinasse (1962) on the one hand, and Binder and his colleagues (1962) on the other, obtained results which, at first sight, appear dissimilar. Espinasse (1962) at Hull University, working in anurans, found the refractive index of the lenses and the optical functioning to be much more normal in the presence of foetal cytolysing tissue than in the controls. Binder *et al.* (1962), working on the influence of embryonic implants upon lens regeneration in adult rabbits, found that eye-lid ectoderm from 16 day old embryos did not promote lens regeneration. This difference in finding may be due to the different source of foetal implant. Espinasse used neurilemma, which would contain both ectodermal and mesodermal material, and this may, therefore, explain his positive results as compared with the negative findings of Binder.

As it appears that the process of tissue differentiation is one of the modulating forces which is lost in carcinogenesis, and that there is a progression towards an increasingly anaplastic cell, it is necessary to consider what is known of the forces controlling tissue growth and organisation. These may be divided into locally operating, and systemic circulatory forces which are probably hormonal.

Rosenberg (1964) grew human conjunctival cells at an interface between two liquids, one hydrophilic and the other hydrophobic, and found the cells grew in layers, the shape of which varied according to the liquids. When he injected lecithin between the layers, however, the cells grew in clumps. Waddington (1959) also grew kidney cells on glass, and obtained epithelial sheets without tubule formation. When, however, he added cholesterol, which prevented adhesion of the cells to the glass, tubules were formed. He believes that cells need to be in certain configurations to function properly, and that mesoderm contains a factor which prevents adhesion of the cells to glass surfaces.

The work of Sanford suggests that when cells are removed from local and systemic controlling forces for long periods of time in serial tissue culture, there is a tendency for malignant change to take place after a period of dedifferentiation has occurred. Differentiation appears to be potentiated by locally acting mesodermal substances which appear to be of a soluble nature, or at any rate, capable of diffusing through a Millipore filter. There is evidence too that both lecithin and cholesterol can initiate a clumping of like cells to like by an unknown mechanism.

Further evidence of the regulatory effect of mesodermal material has recently been given by Pashley and Mandl (1965), who have isolated, by trypsin digestion and fractional precipitation, two components from aorta, tendon and muscle. One of these extracts was found to stimulate growth of normal fibroblasts in tissue culture and the other to inhibit growth of these cells. The growth-inhibitor fraction was also found to inhibit growth of ovarian adenocarcinoma in tissue culture. Anderson and Green (1963) have suggested that tissue specific antigens (TSA), first postulated by Green (1954) are capable of undergoing changes in solubility. It is further suggested that these tissue specific antigens are essential to growth and maintenance of normal organs and that in the intact and healthy organ TSA is in an insoluble form. When, however, organ-cells become displaced from their normal environment, TSA is altered into a soluble form which alerts the disposal mechanisms of the body. Wandering cells which do not form organ-structures are not believed to contain TSA, and malignancies arising in these cells are believed to be due to a break, at a different level, of the morphological control of the cell. If, as we believe, the essential lesion in carcinogenesis is at, or in, the cell surface, and if the living cell is the dynamic organism which we believe it to be, than any agent breaking the chain of command between the gene or genes responsible for the integrity of the cell surface, and that surface, may initiate carcinogenesis. The malignancies of non-TSA bearing cells, the so-called wandering cells of the body, would appear to be of this nature.

### SUMMARY

It is postulated that normal cellular control is maintained by both locally-acting forces and systemic circulatory forces, and both these forces exert their effect in, or on, the cell surface. It is further suggested that the interrelationship between ectoderm and mesoderm, and the solubility or insolubility of tissue specific antigens, exerts the local control.

### REFERENCES

ANDERSON, M. R. AND GREEN, H. N.—(1963) *Nature, Lond.*, **198**, 861.
BINDER, H. F., BINDER, R. F., WELLS, A. H. AND KATZ, R. L.—(1962) *Br. J. Ophthal.*, **46**, 416.
BULLOUGH, W. S. AND RYTÖMAA, T.—(1965) *Nature, Lond.*, **205**, 573.
BURWELL, G. R.—(1963) *Lancet*, ii, 69.
BREMER, H.—(1962) *Wilhelm Roux Arch. EntwMech. Org.*, **154**, 103.
CHAMBERS, R. AND CHAMBERS, E. L.—(1961) 'Explorations into the nature of the living cell'. Cambridge, Mass. (Harvard University Press).
CHANTURISHVILI, P. S.—(1958) *Trans. ophthal. Soc. U.K.*, **78**, 411.
DUBEY, P. N.—(1962) *J. anat. Soc. India*, **11**, 24.
EARLE, W. R., SANFORD, K. K., EVANS, V. J. AND WALTZ, H. N.—(1951) *J. natn. Cancer Inst.*, **12**, 133.
ESPINASSE, P. G.—(1962) *Expl Eye Res.*, **1**, 466.
FIORAMONTI, M. C., EVANS, V. J. AND EARLE, W. R.—(1958) *J. natn. Cancer Inst.*, **21**, 579.
GOSS, R. J.—(1964) 'Adaptive Growth'. London and New York (Academic Press).
GREEN, H. N.—(1954) *Br. med. J.*, ii, 1378.—(1958) *Br. med. Bull.*, **14**, 101.
GROBSTEIN, C.—(1964) *Science, N. Y.*, **143**, 643.
GURDON, J. B.—(1964) *Adv. Morphogenesis* **4**, 21.
LE DOUARIN, N.—(1963) *C. r. hebd. Séanc. Acad. Sci., Paris*, **257**, 255.
LEDUC, E. H. AND WILSON, J. W.—(1963) *J. natn. Cancer Inst.*, **30**, 85.
PACE, D. M. AND AFTONMAS, L.—(1957) *J. natn. Cancer Inst.*, **19**, 1065.
PARDEE, A. B.—(1963) *Natn. Cancer Inst. Monogr.*, **14** 7.
PASHLEY, M. S. AND MANDL, I.—(1965) *Nature, Lond.*, **208**, 800.
ROSENBERG, M. D.—(1964) 'Cellular control mechanisms and cancer', edited by Emmelot, P. and Mühlbock, O. Amsterdam (Elsevier Press).
SACHS, L.—(1964) 'New perspectives in biology', edited by Sela, M. Amsterdam (Elsevier Press), Vol. 4, p. 263.
SACHS, L. AND FOGEL M.—(1964) *Expl Cell Res.*, **34** 448.
SANFORD, K. K.—(1965) *Int. Rev. Cytol.*, **18**, 249.
SANFORD, K. K., EARLE, W. R. AND LIKELY, G. D.—(1948) *J. natn. Cancer Inst.*, **9**, 229.
SANFORD, K. K., MERVIN, R. M., HOBBS, G. L., FIORAMONTI, M. C. AND EARLE, W. R.—(1958) *J. natn. Cancer Inst.*, **20**, 121.

Shelton, E., Evans, V. J. and Parker, G. A.—(1963) *J. natn. Cancer Inst.*, **30**, 377.
Singer, M. and Mutterperl, E.—(1963) *Devl Biol.*, **7**, 180.
Thornton, C. S.—(1962) *J. exp. Zool.*, **130**, 3.
Thornton, C. S. and Steen, T. P.—(1962) *Devl Biol.*, **5**, 328.
Waddington, C. H.—(1959) 'Biological Organisation'. Symposium organised on behalf of U.N.E.S.C.O. by C. H. Waddington. London (Pergamon Press).
Weiler, E.—(1959) *Expl Cell Res.* (Suppl.), **7**, 244.
Weiss, P.—(1960) *Int. Rev. Cytol.*, **9**, 187.
Weiss, P. and Kavanau, J. L.—(1957) *J. gen. Physiol.*, **41**, 1.